# Admission Glucose Levels May Increase the Risk for Early Neurological Deterioration in Females With Acute Ischemic Stroke

**DOI:** 10.3389/fneur.2020.548892

**Published:** 2020-11-05

**Authors:** Zhi-Xin Huang, Yan Huang, Jie Zeng, Hong Hao, Greg F. Petroski, Haike Lu, Xintong Liu, Zhenguo Liu

**Affiliations:** ^1^Department of Neurology, Guangdong Second Provincial General Hospital, Guangzhou, China; ^2^Department of Neurology, The Second School of Clinical Medicine, Southern Medical University, Guangzhou, China; ^3^Center for Precision Medicine and Division of Cardiovascular Medicine, Department of Medicine, University of Missouri School of Medicine, Columbia, MO, United States; ^4^Center for Clinical Epidemiology and Methodology, Guangdong Second Provincial General Hospital, Guangzhou, China; ^5^Biostatistics and Research Design Unit, University of Missouri School of Medicine, Columbia, MO, United States

**Keywords:** early neurological deterioration, glucose, ischemic stroke, risk factor, sex difference

## Abstract

**Background and purpose:** Early neurological deterioration (END) is associated with poor outcome for patients with acute ischemic stroke (AIS). Patients with hyperglycemia have increased risk for stroke and tend to have poor outcome with and without diabetes after stroke. The present study aimed to determine if blood glucose was associated with END and if sex difference was present in the development of END in AIS patients.

**Methods:** A total of 220 consecutive patients (both males and females) with AIS between 2012 and 2015 were screened for this retrospective study. After exclusion, 213 patients were included for analysis. Propensity-score matching was used for normalization of variables including stroke severity, time from symptom onset to treatment, and treatment methods.

**Results:** END was present in 68 patients (31.9%). Multivariate regression analysis showed that the risk of END was significantly higher in males with AIS than in females (*P* < 0.001), and admission blood glucose level was independently associated with END (*P* < 0.001). However, subgroup analysis demonstrated that admission glucose levels were significantly associated with increased risk for END only in females, but not in males (*P* = 0.008). When the cutoff value of 107.1 mg/dL was used, the admission blood glucose level had a significant predictive value for END prediction with a sensitivity of 100% and a specificity of 53% in female patients.

**Conclusions:** The data demonstrated that sex difference was present for the development of END in AIS patients with an increased risk for males. The present study also showed that admission glucose level could be an important predicting factor for END in female patients with AIS.

## Introduction

Stroke is the main cause of mortality and long-term disability worldwide (1). In China, stroke burden has substantially increased over the last 30 years with ~70% of strokes as ischemic in nature (2). Therapeutic strategies including endovascular treatment (EVT) or intravenous recombinant tissue plasminogen activator (IV rt–PA) bridging EVT have improved revascularization rate and functional outcome in patients with acute ischemic stroke (AIS) who received recanalization treatment within 6 h after onset of symptom (3, 4). However, a considerable number of patients develop early neurological deterioration (END) and do not recover from AIS with successful and timely recanalization (5). Because END is closely related to a poor prognosis (6), preventing and treating END are critical to optimal recovery with improved outcome for patients with AIS.

However, the mechanisms for END have been largely undefined, thus limiting the effective prevention and treatment of END. Previous studies have shown that END in the acute phase of AIS was associated with the severity of stroke on admission as measured by the National Institutes of Health Stroke Scale (NIHSS) (7), treatment delay (8), and less optimal treatment strategies (9, 10). Although the EVT techniques have been well-developed and implemented broadly in major stroke centers in the world in recent years with short door-to-needle time (DNT) and improved patient outcome with AIS, END remains highly unpredictable and insidious clinically.

It is known that patients with hyperglycemia have increased risk for stroke especially ischemic stroke (11). Elevated levels of blood glucose in the periinfarct period are closely associated with poor outcome in patients with AIS (12). Clinical studies suggested that there might be a sex difference in the response to treatments and clinical outcomes for patients with AIS. However, it is unclear if elevated blood glucose at the initial presentation of stroke could have a significant impact on END for stroke patients with AIS. The present study aimed to determine if admission blood glucose was associated with END in patients with AIS and successful recanalization treatment and to determine if there was a sex difference.

## Methods

### Study Subjects

The retrospective study protocol was reviewed and approved by the institutional ethical committee. After removal of all personal identifiable information, the medical records of 220 consecutive patients with AIS who received recanalization treatment (EVT after IV rt-PA, EVT, or IV rt-PA alone) in our center between January 2012 and September 2015 were reviewed and analyzed. Without delaying intravenous thrombolysis, all the patients underwent a computed tomography perfusion (CTP) combined with computed tomography angiography (CTA) scan, including non-contrast CT. Patients were excluded from the study if the imaging evidence showed the presence of hemorrhage, aneurysm, arteriovenous malformation, or early ischemic areas exceeding 1/3 of the middle cerebral artery territory on non-contrast CT.

All the patients received either the standard intravenous thrombolysis alone or EVT (including IV rt–PA bridging EVT) if the strokes involved the internal carotid artery (ICA), middle cerebral artery segment M1 and M2, or basilar artery (BA) occlusions (large artery occlusion) as confirmed by CTA. All AIS patients with large artery occlusion within 4.5 h after symptom onset were thrombolyzed intravenously with rt-PA and were prepared to undergo digital subtraction angiography (DSA) for EVT. Additional EVT was performed on patients who had persistent occlusion of the large artery upon follow-up DSA after rt-PA treatment. EVT alone was performed in patients with large artery occlusion of the anterior circulation 4.5–8 h after symptom onset or a posterior circulation occlusion 4.5–24 h after symptom onset. A complete medical history was obtained for each patient with the inclusion of major co-confounding cardiovascular risk factors for the study (13). The demographic data and baseline clinical characteristics were retrospectively reviewed. The patients were divided into the non-END and END groups according to their early neurological status for analysis.

### Definition of END

After admission and recanalization treatment, the patients were evaluated using NIHSS score by two neurologists separately who were blinded to the patient's information and radiographic images. When inconsistent scores came up from the two evaluators, a consensus was reached through discussion. In the present study, END was defined as an increase in the NIHSS score by 2 or more points within 72 h of admission. This criterion for END was used since it could be minimally subject to inter-evaluator variations and could distinguish END from late deterioration mainly due to stroke complications, such as aspiration pneumonia or recurrent stroke (14).

### Statistical Analysis

Continuous variables with a normal distribution were expressed as mean ± standard deviation (SD), variables with non-normal distribution were presented as median [interquartile range (IQR)], and categorical variables were expressed as frequency (percentages). To reduce the impact of heterogeneity (NIHSS score on admission, treatment delay, and treatment methods), propensity-score matching (PSM) was performed to compare influencing factors between the END and non-END groups. In short, one-to-one PSM without replacement (the nearest neighbor technique with a caliper of 0.1) was adopted to minimize the bias from inadequate randomization and potential heterogeneity of AIS patients. We compared the pre- and post-matched patient baseline characteristics using independent sample t and Mann–Whitney U-tests for continuous variables and the chi-squared and Fisher's exact tests for categorical variables as appropriate. PSM was performed by unconditional logistic regression to control for variables (stroke severity, treatment delay, and treatment methods) that were known to have significant impact on END. The present study was performed to identify new factors that could contribute to the development of END.

After matching, the data were analyzed with a binary logistic regression model (forward stepwise method) for all factors with a *P* < 0.1 [sex, body mass index (BMI), glucose, high-density lipoprotein (HDL), low-density lipoprotein (LDL), total cholesterol, diabetes, and hypertension] by univariate analysis to determine their potential relation to END, and the goodness-of-fit of the models was assessed using the Hosmer–Lemeshow test. C-statistics was then used to evaluate the discriminatory and calibration ability of the binary logistic regression model. Finally, univariate and multivariate regression analyses were conducted to identify significant predictive variables associated with END in the male and female groups.

The sensitivity and specificity for a variety of points on receiver operating characteristic (ROC) curves were obtained. The optimal cutoff value for END was determined using Youden's index (J = Sensitivity + Specificity – 1). The 95% confidence interval (CI) of an END diagnosis was obtained using the Wilson method. A two-tailed *P*-value of 0.05 or less was considered statistically significant. All statistical analyses were performed with SPSS software, version 25.0 (SPSS Inc., Chicago, IL, United States).

## Results

### Patient Demographic and Clinical Characteristics

**Figure 1:**
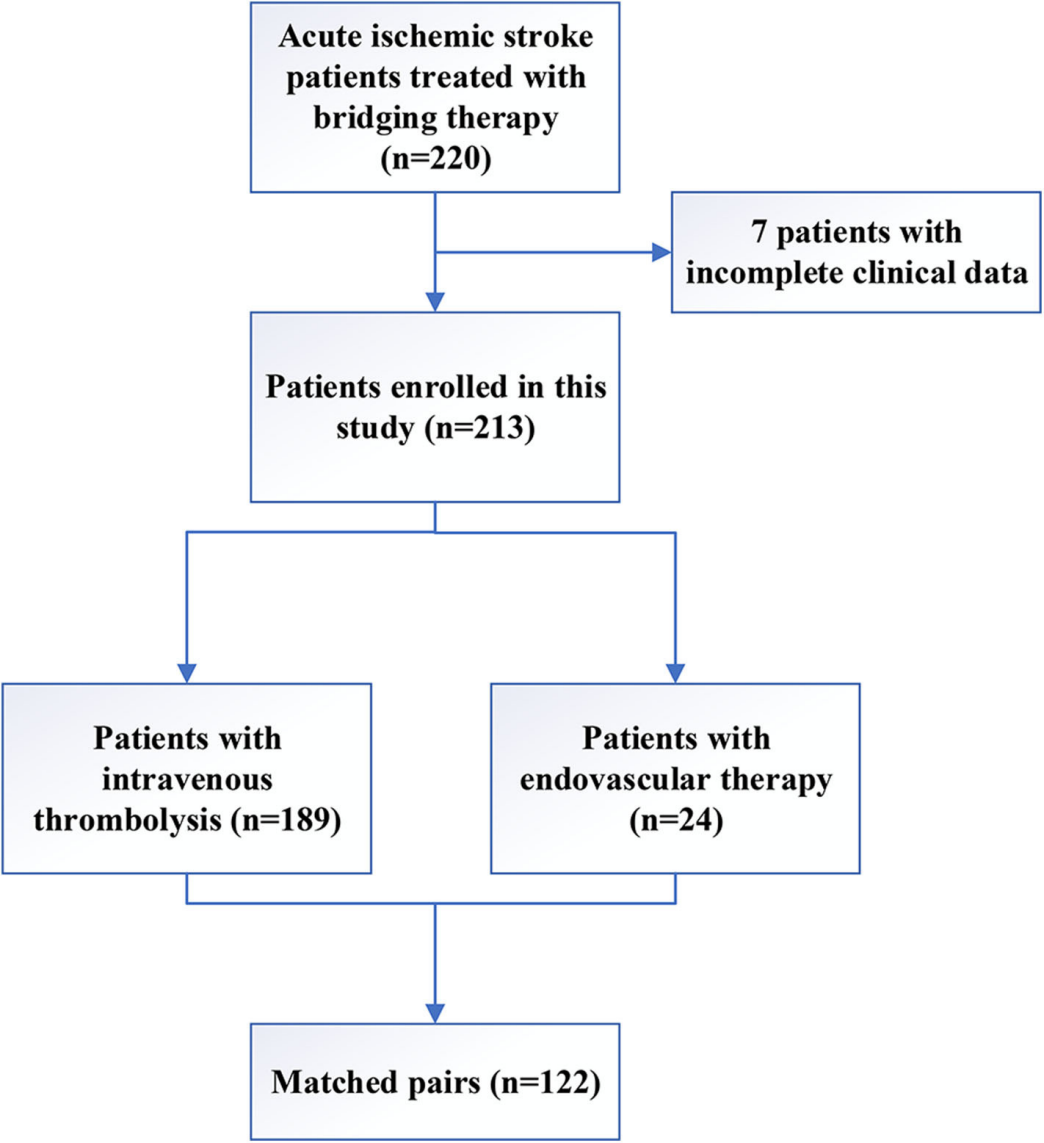
Flowchart showing study participant selection.

A total of 220 AIS patients who underwent recanalization therapy in our stroke center between 2012 and 2015 were screened for the study, and 7 patients were excluded due to incomplete clinical data (Figure 1). The remaining 213 patients were included in this study, including 189 patients who underwent IV rt-PA and 24 (11.3%) patients who received EVT. The mean age of the patients was 60.9 years, and 101 (47.4%) patients were females. During hospitalization, 68 (31.9%) patients suffered from END. The median time from sign or symptom onset to treatment was 3.3 h (IQR 1.9), and the median NIHSS score at admission was 10 (IQR 6.0).

### Baseline Characteristics Before and After PSM

**Figure 2:**
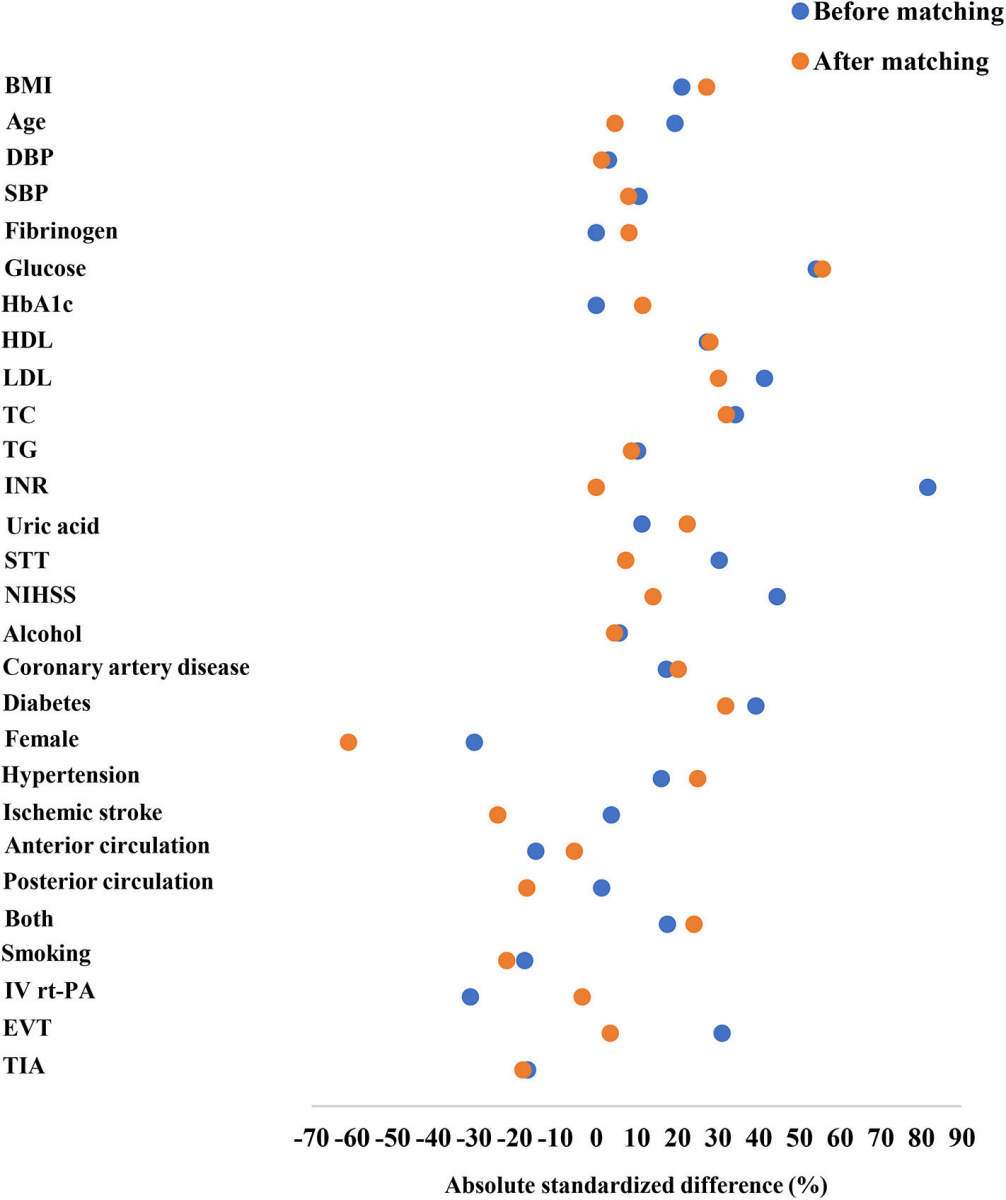
Absolute standardized differences in the baseline characteristics of AIS patients after recanalization therapy before and after 1:1 propensity-score matching were shown. The Y axis showed the baseline characteristics, and the scatterplot represented the status before or after matching.

Patient characteristics before and after matching are shown in Table 1 and Figure 2. Before matching, glucose, total cholesterol, HDL, and LDL levels in patients with END were significantly higher than those in the non-END group (*P* < 0.05). Diabetes was more prevalent in the END group (29.4%) than in the non-END group (9.7%) (*P* < 0.001). The percentage of female patients was significantly lower in the END group (35.3%) than in the non-END group (53.1%) (*P* = 0.015). Significant differences in the NIHSS score on admission, time from symptom onset to treatment, and treatment methods were present between the two groups (*P* < 0.05). After matching, no statistically significant differences were observed between the two groups (61 patients in each group) in total cholesterol levels, NIHSS scores, time from symptom onset to treatment, and treatment method; however, significant differences in diabetes, percentage of female patients, and glucose, HDL, and LDL levels persisted between the two groups (Table 1).

**Table 1:** Patients' clinical and demographic characteristics before and after propensity-score matching.

	**Before propensity-score matching**			**After propensity-score matching**		
**Characteristic**	**END**	**Non-END**	***P*-value**	**END**	**Non-END**	***P*-value**
No. of patients	68	145	–	61	61	–
**Demographics**						
Age, year	62.9 ± 12.9	60.0 ± 10.6	0.080	61.6 ± 12.7	60.9 ± 11.3	0.777
Female, *n* (%)	24 (35.3)	77 (53.1)	0.015	17 (27.9)	38 (62.3)	<0.001
Smoking, *n* (%)	8 (11.8)	28 (19.3)	0.172	8 (13.1)	14 (23.0)	0.158
Alcohol intake, *n* (%)	6 (8.8)	10 (6.9)	0.620	6 (9.8)	5 (8.2)	0.752
BMI, kg/m^2^	25.2 ± 4.3	24.1 ± 4.2	0.077	25.3 ± 4.3	23.9 ± 4.0	0.062
DBP, mm Hg	92.1 ± 20.8	91.4 ± 13.8	0.822	93.6 ± 20.4	93.3 ± 13.4	0.328
SBP, mm Hg	153.4 ± 27.4	150.1 ± 21.6	0.348	152.8 ± 24.6	150.5 ± 21.2	0.597
**Laboratory measures**						
Fibrinogen, g/L	3.2 ± 1.1	3.2 ± 0.8	0.580	3.4 ± 1.1	3.3 ± 0.8	0.638
Glucose, mg/dL	174.6 ± 97.2	120.6 ± 30.6	<0.001	180.0 ± 108.0	118.6 ± 28.8	<0.001
Hemoglobin A1C, %	7.3 ± 0.8	7.3 ± 0.5	0.518	7.4 ± 0.8	7.3 ± 0.5	0.370
HDL, mmol/L	1.4 ± 0.7	1.2 ± 0.3	0.005	1.4 ± 0.7	1.2 ± 0.2	0.035
LDL, mmol/L	3.7 ± 1.3	3.1 ± 0.9	0.003	3.7 ± 1.5	3.2 ± 1.0	0.049
Total cholesterol, mmol/L	5.5 ± 1.6	4.9 ± 1.0	0.008	5.6 ± 1.7	5.0 ± 1.1	0.056
Triacylglycerol, mmol/L	1.5 ± 0.8	1.4 ± 0.8	0.666	1.5 ± 1.0	1.4 ± 0.8	0.524
INR	1.1 ± 0.1	1.0 ± 0.1	0.877	1.0 ± 0.1	1.0 ± 0.1	0.678
Uric acid, μmol/L	305.2 ± 91.2	293.5 ± 70.0	0.302	311.6 ± 92.3	288.3 ± 68.1	0.116
**Medical history**						
Coronary artery disease, *n* (%)	22 (32.4)	33 (22.8)	0.137	22 (36.1)	15 (24.6)	0.168
Diabetes mellitus, *n* (%)	20 (29.4)	14 (9.7)	<0.001	18 (29.5)	8 (13.1)	0.027
Hypertension, *n* (%)	44 (64.7)	80 (55.2)	0.190	40 (65.6)	31 (50.8)	0.099
Ischemic stroke, *n* (%)	10 (14.7)	19 (13.1)	0.689	6 (9.8)	12 (19.7)	0.135
**Clinical characteristics**						
Offending vessel			0.145			0.184
Anterior circulation, *n* (%)	38 (55.9)	94 (64.8)		37 (60.7)	39 (63.9)	
Posterior circulation, *n* (%)	16 (23.5)	33 (22.8)		10 (16.4)	15 (24.6)	
Both, *n* (%)	14 (20.6)	18 (12.4)		14 (23.0)	7 (11.5)	
STT, h, median (IQR)	4.0 (3.0–5.0)	3.0 (2.5–4.5)	0.003	3.7 (3.0–4.5)	3.5 (2.6–5.0)	0.471
NIHSS, median (IQR)	12.0 (10.0–18.0)	10.0 (7.0–12.0)	<0.001	12.0 (10.0–15.5)	11.0 (7.0–16.0)	0.241
Treatment methods			0.003			0.810
IV rt-PA	54 (79.4)	135 (93.1)		50 (82.0)	51 (83.6)	
EVT	14 (20.6)	10 (6.9)		11 (18.0)	10 (16.4)	

*BMI, body mass index; Both, involvement of the anterior and posterior circulation; DBP, diastolic blood pressure; EVT, endovascular treatment; HDL, high-density lipoprotein; INR, International Sensitivity Index; IQR, interquartile range; IV rt-PA, intravenous recombinant tissue plasminogen activator; LDL, low-density lipoprotein; NIHSS, National Institutes of Health Stroke Scale; SBP, systolic blood pressure; STT, time from symptom onset to treatment*.

### Association Between END and Blood Glucose

After matching, a binary logistic regression analysis was performed to identify the factors that might be associated with END in patients with AIS after recanalization treatment. Compared with patients without END, patients with END had significantly higher initial glucose levels [odds ratio (OR), 1.03; 95% CI, 1.01–1.04; *P* < 0.001].

### Sex Differences in the Development of END

**Figure 3:**
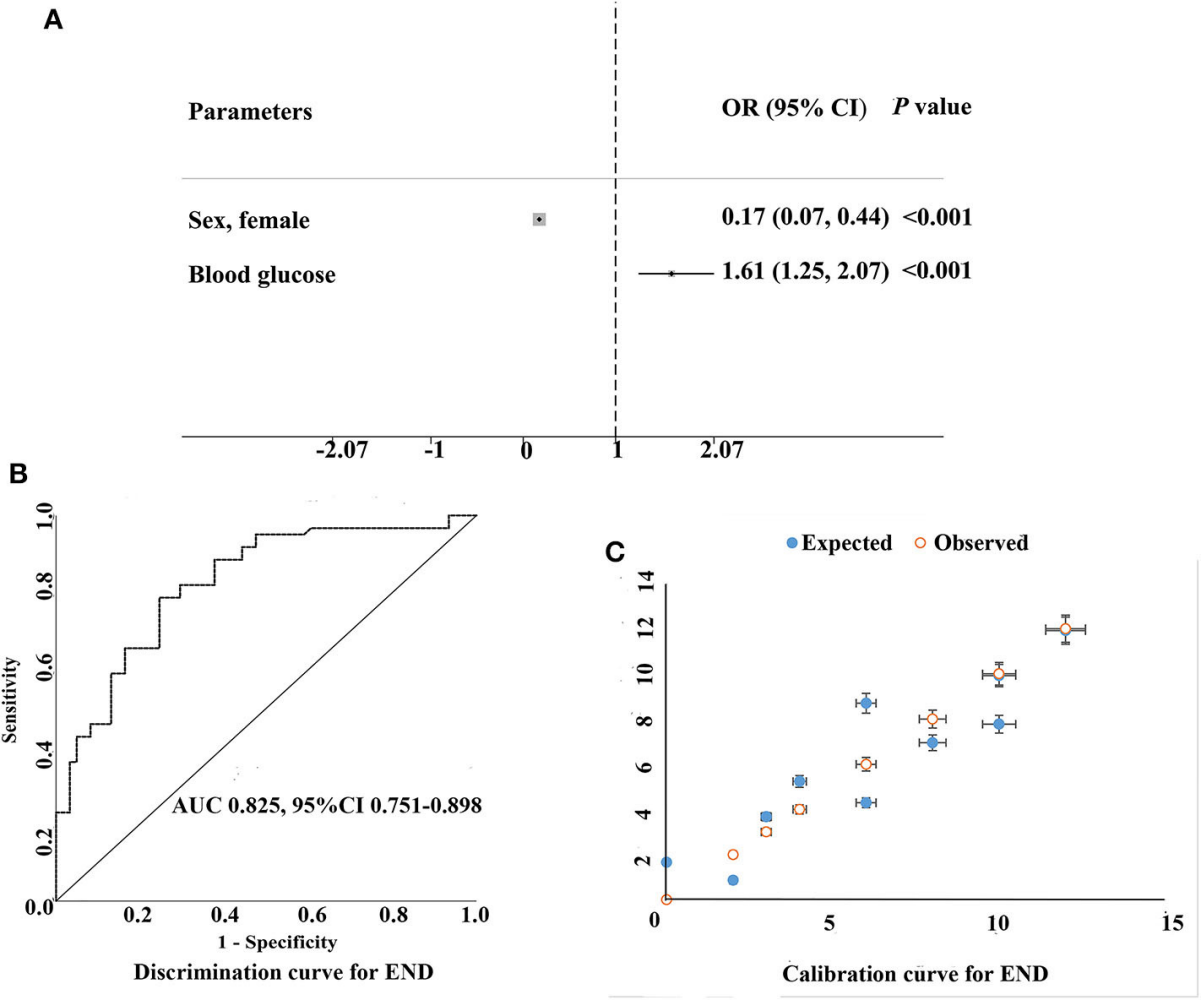
Multivariable logistic model on identifying the predictors of early neurological deterioration following PSM matching. **(A)** Multivariable-adjusted odds ratios (diamonds) and 95% confidence intervals (CI; bars) for risk factors of early neurological deterioration were shown. **(B,C)** show the discrimination and calibration of the prediction model respectively, for example, the AUC of the area under the ROC curve is 0.825 > 0.75 and the 95% CI is 0.751−0.898, suggesting that the prediction model has good discrimination ability. **(C)** shows the consistency between the predicted risk of the model and the actual observed occurrence risk.

**Figure 4:**
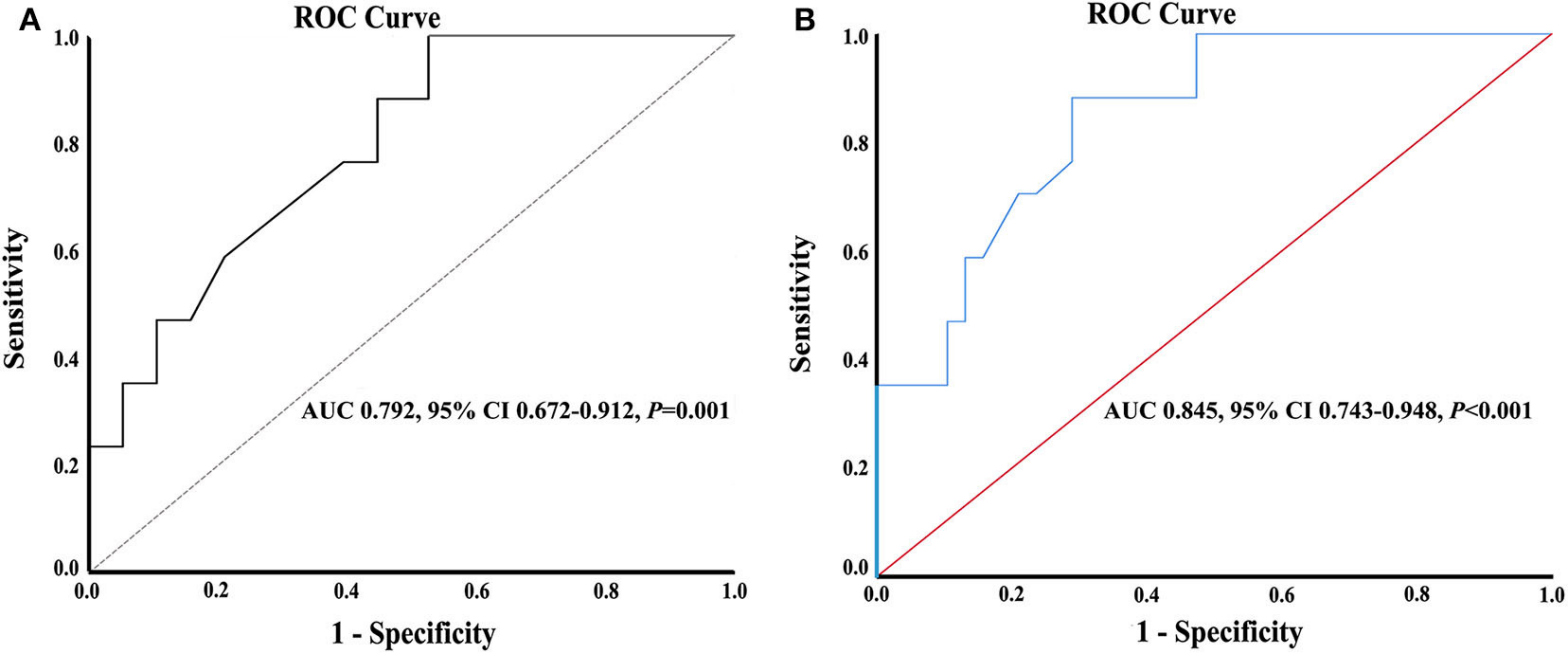
**(A)** Receiver operating characteristic curves with the corresponding areas under the curves for glucose in predicting early neurological deterioration in female patients with acute ischemic stroke. **(B)** Discrimination curve for END.

Significant variables in the univariate analysis were included in the multivariate logistic regression analysis. After adjustment for other confounding factors (smoking, diabetes, total cholesterol, HDL, LDL, and BMI), women with AIS had decreased risk for END as compared with men (OR, 0.17; 95% CI, 0.07–0.44; *P* < 0.001), and the area under the curve (AUC) of discrimination was 0.825 (Tables 1, 2, Figure 3). LDL level and BMI at admission were higher in men than in women (3.7 ± 1.5 vs. 3.2 ± 1.0, *P* = 0.019, and 25.4 ± 4.2 vs. 23.7 ± 4.0, *P* = 0.028, for LDL and BMI in men and women, respectively). The glucose level in female patients (*n* = 55) was associated with increased risk for END (OR, 1.04; 95% CI, 1.01–1.06; *P* = 0.008). However, in the stepwise binary regression analysis for the association with END in men (*n* = 67), no variables were statistically significant (*P* > 0.05). The cutoff point on the ROC analysis showed significant predictive power for glucose as a risk for END in women as shown in Figure 4A. The plot indicated excellent prediction for glucose with an AUC of 0.792 (95% CI = 0.672–0.912, standard error = 0.061, *P* = 0.001). The area under the ROC curve was 0.845 (95% CI, 0.743–0.948; *P* < 0.001), suggesting an adequate discrimination for female patients who could have or not have END (Figure 4B).

**Table 2:** Multivariate logistic regression analysis for END following PSM matching.

**Variable**	**OR (95% CI)**	***P*-value**
Glucose	1.03 (1.01–1.04)	<0.001
Sex, female	0.18 (0.07–0.45)	<0.001
Male	Reference	
HDL	3.52 (0.85–14.56)	0.082

*CI, confidence interval; END, early neurological deterioration; OR, odds ratio; PSM, propensity-score matching*.

## Discussion

Although revascularization therapy is a safe and effective treatment for patients with AIS, many patients still suffer from END after recanalization therapy. Many factors are associated with poor neurological outcomes, such as treatment delay, poor NIHSS score at admission, and less optimal treatment methods. After matching these three factors, we found that sex, specifically males, and glucose level were significantly correlated with END. To the best of our knowledge, this is the first study demonstrating that sex and hyperglycemia are associated with END in Chinese patients with AIS after revascularization therapy. Moreover, elevated blood glucose is an independent risk factor for END in female patients.

Of note, the criteria to define END have been inconsistent in terms of the degree of neurological deficits or the timeframes in clinical studies and are still evolving (15). Some studies defined END as ≥4–point increase in NIHSS between admission and 24 h (16). The NIHSS puts significant amount of considerations on motor dysfunction, whereas some non-motor, and yet important, functions, such as neglect, are underweighted (17). Thus, NIHSS may have significant limitations on the evaluation for patients with END. For instance, a functionally severe worsening of neglect may be counted as 2 points only, which would not meet the criteria as END using the ΔNIHSS ≥4 definition. Recently, END has been defined as ≥1–point increase in motor NIHSS or ≥2–point increase in total NIHSS (18, 19), or ≥2–point increase in NIHSS between admission and 24 h (20), and ≥2–point increase in NIHSS within 72 h (14). One of the advantages of a conservative END definition (for example, an increase in NIHSS of ≥2 points) could be an early identification of primary neurological damages with or without effective interventions in patients with AIS. One study showed that increased death and major disability were observed in patients with increases in NIHSS of ≥2 points (21). In contrast, a more liberal definition (an increase in NIHSS of ≥4 points) would be advantageous for minimizing work-up costs. Because of the different definitions of END, the reported rate of END occurring after interventional therapy varied greatly in clinical studies. In the present study, END was defined as an increase in the NIHSS score of 2 or more points within 72 h of admission (14, 22). This criterion for END was used since it could minimize inter-evaluator variations and could distinguish END from late deterioration mainly due to stroke complications, such as aspiration pneumonia or recurrent stroke.

### Association Between Sex and END

In the present retrospective study of hospitalized patients with AIS, we found that 31.9% of the patients experienced END in the acute phase, which is in accordance with previous studies (23). The incidence of END after AIS varied considerably and is associated with various factors including stroke severity, blood pressure, and genetic factors, etc. (24). In the present study, we observed that male patients with AIS were more prone to END, which may be attributed to several mechanisms. It was reported that decreasing the production of free hydroxyl radicals and increasing the activity of antioxidant enzymes could significantly reduce acute cerebral ischemic brain damage (25). There are clear differences in the expression and/or activities of some antioxidant enzymes between males and females (26). Thus, the activities of catalase (the enzyme involved in the degradation of hydrogen peroxide) and superoxide dismutase (SOD) in peritoneal macrophages in male rats are significantly lower than those in female rats (26). Sex-related differences in antioxidant enzyme activity are associated with differences in circulating sex hormone levels (27).

Stroke-induced immunodepression has been identified in clinical and experimental studies and considered to be associated with lymphopenia and T cell dysfunction (28, 29), leading to increased risk for infection after stroke and worsening neurological deterioration (30). Estrogen may directly regulate the post-stroke immune response through sex steroid receptors in the cells (including microglia, astrocytes, and various circulating immune cells) in the brain (31). Animal study showed that, compared with age-matched male animals, circulating estrogen could significantly reduce ischemic lesions in females (32).

The finding that sex difference exists for the development of END in patients with AIS has clear and important clinical significance. It could help to better understand the differences in functional outcomes between women and men after recanalization treatment and to provide appropriate measures for effective prevention of END for high-risk patients.

One may argue that the number of women who developed END was small (a total of 17) in the present study, thus not allowing to draw a meaningful conclusion. Indeed, END was observed in a small number of women with AIS in the present study. However, based on the rule of 10 events per variable (33), the sample size for the present study might be appropriate to support a valid conclusion, because there was only one variable of glucose in the model, and the area under the ROC curve was 0.845 (95% CI, 0.743–0.948; *P* < 0.001), suggesting an adequate discrimination for patients who could have or not have END (34–36). Certainly, a multi-center study with large patient size is needed to confirm the findings in the present study with high statistical power and with incorporation of other important variables including (but not limited to): (1) rates of ICH, (2) baseline ASPECTS, (3) perfusion mismatch, (4) type of lesion suspected (atherosclerotic or embolic), and (5) EVT (stent retriever/aspiration or both) and subsequent thrombolysis in cerebral infarction (TICI) results, to better understand the influence of these factors on END in the patients.

### Association Between Hyperglycemia, END, and Sex

In the present study, we found that glucose levels at admission were higher among patients with END than those without END (10.0 ± 6.0 vs. 6.6 ± 1.6, *P* < 0.001). Multivariate analysis suggested that hyperglycemia was correlated with END in patients with AIS after adjusting for other confounding factors. Hyperglycemia is common in acute stroke patients with or without diabetes, as a result of acute stress response (also commonly known as stress hyperglycemia) (37). Subgroup analysis showed that when the glucose cutoff value of 107.1 mg/dL was used for predicting END, the admission glucose level has significant predictive value with a sensitivity rate of 100% and a specificity rate of 53% in female patients, but not in males. A previous study reported that hyperglycemia, which was defined as blood glucose ≥154.8 mg/dL, at any time within the first 48 h after stroke was associated with a poor prognosis regardless of the infarct size, presence of diabetes, stroke severity, or age (38). The mechanisms of increased risk for END associated with hyperglycemia may include endothelial injury, active oxygen species production, blood–brain barrier destruction, and tissue acidosis (39–42).

In addition to hypoglycemia and hyperglycemia, glycemic variability (GV) was considered as another important component of dysglycemia (43). It was reported that GV within the initial 3 hospital days was associated with increased risk for END in diabetic patients with AIS (44). Although the mechanism for the occurrence of GV in patients with AIS is not clear, GV was strongly associated with infarct volume, severity of symptoms, and stroke outcomes (45, 46). It could be important clinically to determine if the patients with elevated blood glucose levels at admission could have increased risk for GV in diabetic patients and to determine if a combined approach of optimal glycemic control and minimizing glucose fluctuations in diabetic patients could improve the outcome for stroke patients in the future studies.

It is unclear why hyperglycemia was associated with END in women, but not in men. The possible reasons could include: (1) compared with men, women have a higher prevalence of impaired glucose tolerance and lower insulin sensitivity (47), and (2) metabolic syndrome (including hyperglycemia) is more frequent in women with AIS (48). This finding underscores the positive correlation between admission glucose levels and END in AIS women with blood glucose levels above 107.1 mg/dL at admission. However, further studies are needed to determine if optimal control of blood glucose (with fast-acting insulin for example) could enhance the clinical outcome for women with AIS.

## Limitations

Several potential limitations to this study exist. First, this is a retrospective single-center study with patients of one specific ethnic background; therefore, our findings may not be broadly applicable. Second, although the sample size for the present study was appropriate according to the rule of 10 events per variable (33), the female-specific subset in the present study was relatively small, with only 17 women experiencing END, although a statistically significant association was present between admission blood glucose levels and END in female patients. A multi-center study with large patient population is needed to confirm the findings in the present study. And third, since the present study excluded stroke patients with larger areas of infarction, the results were more applicable to patients with stroke of smaller infarcts.

## Conclusion

In conclusion, the data from our study demonstrated that sex difference was present in the development of END in patients with AIS with increased risk for males. The present study also showed that admission glucose level was an important factor for prediction of END for female patients with AIS. Further studies are needed to determine if optimal control of initial blood glucose level could prevent END and improve clinical outcome for female patients with AIS.

## Data Availability Statement

The raw data supporting the conclusions of this article will be made available by the authors, without undue reservation.

## Ethics Statement

The studies involving human participants were reviewed and approved by the Ethical Committee of the Guangdong Second Provincial General Hospital. The patients/participants provided their written informed consent to participate in this study.

## Author Contributions

Z-XH, YH, and HH: conception and design of the study. Z-XH, YH, JZ, HH, GP, HL, XL, and ZL: data acquisition and analysis. Z-XH and ZL: drafting and revising the manuscript critically. All authors contributed to the article and approved the submitted version.

## Conflict of Interest

The authors declare that the research was conducted in the absence of any commercial or financial relationships that could be construed as a potential conflict of interest. The reviewer BF declared a shared affiliation with several of the authors, HH and ZL, to the handling editor at time of review.
